# Protein characterization of an Indonesian isolate of foot and mouth disease virus inactivated with formaldehyde and binary ethylenimine

**DOI:** 10.14202/vetworld.2024.1836-1845

**Published:** 2024-08-24

**Authors:** Yudha Kurniawan, Wiwiek Tyasningsih, Jola Rahmahani, Yulianna Puspitasari, Kusnoto Kusnoto, Fadia Azzahra, Talenta Miracle Tobing, Ahmad Aswin, Diyantoro Diyantoro, Firdausy Kurnia Maulana, Helen Susilowati, Suryo Kuncorojakti, Fedik Abdul Rantam

**Affiliations:** 1 Magister Program in Vaccinology and Immunotherapeutic, Faculty of Veterinary Medicine, Universitas Airlangga, Surabaya, East Java, Indonesia; 2 Division of Veterinary Microbiology, Department of Veterinary Science, Faculty of Veterinary Medicine, Universitas Airlangga, Surabaya, East Java, Indonesia; 3 Department of Parasitology, Faculty of Veterinary Medicine, Universitas Airlangga, Surabaya, East Java, Indonesia; 4 Bachelor Program in Veterinary Medicine, Faculty of Veterinary Medicine, Universitas Airlangga, Surabaya, East Java, Indonesia; 5 Research Centre for Vaccine Technology and Development, Institute of Tropical Disease, Universitas Airlangga, Surabaya, East Java, Indonesia; 6 Faculty of Vocational Studies, Universitas Airlangga, Surabaya, East Java, Indonesia; 7 Division of Veterinary Anatomy, Department of Veterinary Science, Faculty of Veterinary Medicine, Universitas Airlangga, Surabaya, East Java, Indonesia

**Keywords:** binary ethylenimine, foot-and-mouth disease virus, formaldehyde, protein

## Abstract

**Background and Aim:**

Foot-and-mouth disease (FMD) is a highly contagious viral disease of cloven-footed animals. It is a major threat to livestock production worldwide, causing significant economic losses. Inactivation of FMD virus (FMDV) is crucial for vaccine development and control of outbreaks. However, traditional inactivation methods can sometimes damage the viral protein, affecting vaccine efficacy. Therefore, finding new inactivating agents that effectively inactivate the virus while preserving the integrity of its proteins is an important research area. This study investigated the optimal materials (0.04% formaldehyde, 0.001 M binary ethylenimine [BEI], or a combination) for inactivating and preserving the specific molecular weight of Serotype O FMDV protein.

**Materials and Methods:**

This study used serotype O FMDV isolated from several areas of East Java. The virus was inoculated into baby hamster kidney-21 cells, and the titer was calculated using the TCID_50_ Assay. The virus was inactivated using 0.04% formaldehyde, 0.001 M BEI, or a combination of 0.04% formaldehyde and 0.001 M BEI. Inactive viral proteins were characterized using sodium dodecyl sulfate-polyacrylamide gel electrophoresis and western blotting.

**Results:**

Serotype O FMDV can be inactivated using 0.04% formaldehyde while preserving specific FMDV proteins, specifically VP0 and VP3 with a molecular weight (MW) of 36 kDa and VP3 with a MW of 24 kDa. Serotype O FMDV can be inactivated by 0.001 M BEI while preserving specific FMDV proteins, specifically VP0 with a MW of 35 kDa, VP3 with a MW of 28 kDa, and VP1 with a MW of 23 kDa. FMDV serotype O can be inactivated using a combination of 0.04% formaldehyde and 0.001 M BEI while preserving specific FMDV proteins, specifically VP0 and VP3 with a MW of 36 kDa and VP3 with a MW of 24 kDa.

**Conclusion:**

This study found that 0.04% formaldehyde, alone or in combination with 0.001 M BEI, was effective for inactivating and preserving the specific molecular weight of Serotype O FMDV protein. The limitation of this study was the inactivations of the virus have not yet been tested for their potency on experimental animals. Further research is warranted to investigate the inactivation kinetics of these materials, including their potency on experimental animals. Additionally, a comparison of the inactivation rates between 0.04% formaldehyde alone and the combination with BEI would help to determine the optimal inactivation agent for future applications.

## Introduction

Foot-and-mouth disease (FMD) is caused by the Aphthovirus virus from the Picornaviridae family, which rapidly spreads and triggers outbreaks in various regions [1]. FMD is caused by a virus from the Picornaviridae family, Aphthovirus, which quickly spreads to new geographic zones and causes outbreaks [1]. The FMD virus (FMDV) has diverse hosts, high genetic variation, seven serotypes (O, A, Asia-1, C, SAT1, SAT2, and SAT3, and more than 100 serosubtypes [2]. The World Organization for Animal Health indicates that FMD has a morbidity rate reaching 100% among affected populations and a mortality rate of 20%–30% for young animals and 1%–5% for adults. Because of its implications for animal health, FMD threatens the national economy and millions of people who depend on animal agriculture [3]. The FMD is endemic in several South-east Asian countries, including Cambodia, Laos, Malaysia, Myanmar, Thailand, and Vietnam, according to the World Health Organization. In 2022, FMD cases will also begin to spread in Indonesia, which has had no FMD cases since 1983 [4, 5].

Knowledge about the disease and how to handle FMD cases is required so that the impacts of an outbreak can be rapidly minimized, one of which is to initiate a vaccination campaign to form herd immunity [6, 7]. A problem with vaccination programs using FMDV inactive vaccines is that cross-protection against the seven existing serotypes is needed. At the same time, cross-protection is only minimal between several subtypes of the same serotype [2]. High compatibility between virus isolates as vaccine seeds and the viruses that cause outbreaks is the most crucial factor in determining the success of vaccination; therefore, it is necessary to develop vaccines using local isolates [8, 9]. Inactivated viral proteins must retain their immunogenic structure for an effective host immune response. An inactivation process is essential to protect the viral proteins VP1, VP2, and VP3 of FMD virus, as they possess immunogenic epitopes that elicit an immune response to create immunity upon antibody binding [10, 11].

In 1960, an inactivated virus vaccine created in baby hamster kidney (BHK) cells utilizing formaldehyde significantly decreased the occurrence of FMD in European nations [8]. Aldehyde groups such as formaldehyde, through alkylation and binding to purine amino and sulfhydryl groups, inactivate viruses by crosslinking proteins [8, 12]. Binary ethylenimine (BEI) inactivates the FMD virus faster and more linearly than formaldehyde [13, 14]. The capsid of FMDV remains intact during BEI inactivation. BEI is recommended as an inactivating material yet, counting the number of viruses inactivated with BEI yields lower results than inactivation with formaldehyde, which yields 65%–71.6% for formaldehyde and 44.2% for BEI [2]. In a previous study, inactivation of the serotype O FMDV using BEI 0.001 M at a temperature of 7°C for 24 h was demonstrated to be effective [15]. In this study, Indonesian isolates of the serotype O FMDV were inactivated using formaldehyde, BEI, or a combination of formaldehyde and BEI to determine which inactivation material was optimal for inactivating local isolates of the FMD virus by examining the cytopathic effects (CPE) value of the virus when it was propagated back in cells and examining the protein integrity of the post-inactivated virus by reviewing the results of the protein molecular weight (MW) measurements in the protein characterization test.

## Materials and Methods

### Ethical approval

The research methodologies, procedures, and usage of experimental animals have been ethically approved by the Animal Care and Use Committee (ACUC) at the Faculty of Veterinary Medicine, Airlangga University, Surabaya, Indonesia (approval number: 2.KEH.101.06.2023).

### Study period and location

This study was conducted from July to September 2023 in a BSL-2 laboratory at the Research Center for Vaccine Technology and Development, Institute of Tropical Disease, Airlangga University, Surabaya, Indonesia.

### Experimental design

This study tested the inactivation ability of formaldehyde 0.04%, BEI 0.001 M and a combination of formaldehyde 0.04% + BEI 0.001 M to inactivate and maintain the weight of virus-specific protein molecules of the Indonesian isolate of serotype O FMDV. The study was divided into three groups: T1: FMDV inactivated with 0.04% formaldehyde; T2: FMDV inactivated with 0.001 M BEI; and T3: FMDV inactivated with a combination of 0.04% formaldehyde and 0.001 M BEI.

The cryopreserved viral inoculum used in this study originated from serotype O FMDV obtained from oral vesicles of naturally-infected cattle in Lamongan, East Java, Indonesia, a region endemic to FMD.

The isolate was incubated with each inactivation agent at 37°C for 24 h with gentle agitation to assess their inactivation effect. A confluent BHK-21 monolayer was inoculated with an inactivated virus and incubated for 3 days, undergoing three passages. Daily observations of CPE were recorded using a Nikon Eclipse Ts2 FL Inverted Diascopic Epi-Fluorescence Microscope with a DS-Qi2 Cam inverted microscope. The Kruskal–Wallis and Mann–Whitney tests were employed in Statistical Package for the Social Sciences (SPSS)^®^ version 23 (IBM Corp., NY, USA) to analyze the CPE scores. The CPE scores were statistically analyzed using Microsoft Excel^®^ 2019 (Microsoft Office, Washington, USA).

### BHK-21 cell culture

The growth medium used in this study included minimum essential medium (MEM) (Gibco™, Thermo Fisher Scientific, USA) with 10% fetal bovine serum (FBS) (Gibco™, Thermo Fisher Scientific), 1% of amphotericin B (Gibco™, Thermo Fisher Scientific), and 1% of Penicillin Streptomycin (1%) (Gibco™, Thermo Fisher Scientific). The medium and reagents were conditioned to room temperature and pre-warmed in a water bath at 37°C. The myotube containing 1 mL of the BHK-21 cell suspension was taken from the –80°C freezer, thawed, and placed into a 15 mL conical tube for centrifugation at 447.2 × *g* for 5 min. The supernatant was discarded, and the cell pellet was resuspended in 10% MEM and placed in a 25 cm^2^ flask previously filled with 10% MEM. The flask was incubated for 24 h at 37°C and 5% CO_2_ [16]. The cells were then observed under a 40× or 100× magnification Nikon Eclipse Ts2 inverted microscope (Nikon Instruments Inc., Tokyo, Japan) to determine confluence and confirm the absence of bacterial and fungal contamination.

BHK-21 cells were adapted through repeated passages or culture from flasks seeded in an earlier phase of this study. BHK cells were passaged by adding 1 mL of Dulbecco’s phosphate-buffered saline (DPBS; Gibco™, Thermo Fisher Scientific) to rinse off the cell monolayer. The monolayer cells were then split from the bottom of the flask by administering 1 mL of trypsin per 25 cm^2^ of the flask surface, and the flask was shaken gently to cover the entire surface of the monolayer cells. The flask was incubated at 37°C in an atmosphere of 5% CO_2_ for 5 min. The cells were then examined again under an inverted microscope to ensure that they were detached and floating.

As many as 1 mL of 10% MEM was added to stop the action of trypsin. Trypsin was removed through centrifugation for 5 min at 2000 rpm. The supernatant was discarded, and the cell pellet was resuspended in 10% MEM. Next, 5 mL of 10% MEM was added to a newly labeled flask, and the cells were placed in the flask. The flask was incubated for 24 h at 37°C in an atmosphere containing 5% CO_2_ [17, 18].

### Foot and mouth disease virus propagation and adaptation

BHK-21 cells that formed monolayers at 70%–80% confluence (after 24–48 h of sub-culture) were selected for propagation with FMDV isolates. The medium from BHK-21 cells was discarded. Then, 1 mL of the stock FMD virus was propagated into BKH-21 cells and spread to cover the monolayer by tilting the cells for 1 min to ensure better interaction. The flask was incubated at 37°C for 1 h to allow the virus to propagate [19].

The cells were incubated in a 37°C incubator with 5% CO_2_ and treated with 8 mL of 5% MEM. An inverted microscope was used daily to observe cell growth exhibiting FMDV CPE characteristics after incubation. The confirmation of FMD virus entry into BHK-21 cells can be inferred from the onset of CPE featuring round/flat cells, disrupted cell junctions, cell death, and release of cell connections from the bottom of the flask. The FMD virus was adapted to BHK-21 cells through three rounds of blind propagation [17].

### TCID_50_ assay

TCID_50_ calculations were initiated by seeding BHK-21 cells on M-96 culture plates. Before seeding, the number of cells required for each well of the plate was calculated. First, cells cultured in flasks were collected by administering trypsin. After administering trypsin, 1 mL of 10% MEM was added to stop the action of trypsin, and the cell monolayer was transferred into a 15 mL conical tube for centrifugation for 5 min at 2000 rpm. The cell pellet was resuspended in 1 mL of 10% MEM. A total of 10 μL cell and medium were mixed with 0.4% trypan blue at a ratio of 1:1 in an Eppendorf tube. A hemocytometer (Neubauer improved counting chamber, Precicolor HBG, Germany) was used to count BHK-21 cells [20]. Cells mixed with trypan blue were placed in the hemocytometer at a concentration of 10 μL under Nikon Eclipse Ts2 inverted microscope (Nikon Instruments Inc., Tokyo, Japan). Stained (viable) and unstained (nonviable) cells were calculated using the following formula to determine cell concentration:


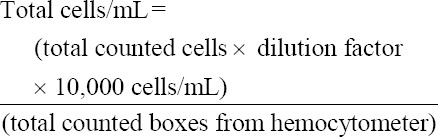


BHK-21 cells were seeded in a 96-well culture plate requires 5 × 10^3^ cells/well. The medium needed for 5 × 10^3^ cells was counted, plated on a culture plate, and incubated for 24 h in a 37°C incubator with 5% CO_2_. Viruses cultured using the method mentioned in FMDV propagation and adaptation paragraph were then harvested. Eppendorf tubes were prepared, and the virus was diluted 10 times from 10^-1^ to 10^-10^ using MEM (–) [minimum essential medium without any added components] and then embedded in M-96 culture plates. The M-96 culture plate was incubated for 1 h to allow the virus to propagate into the cells. The media and viruses in the M-96 culture plates were discarded. Finally, 200 μL/well MEM overlay was added to the M-96 culture plate and incubated at 37°C for 24 h. Cells were observed daily under an inverted microscope to detect plaque formation [21].

On day 5, the MEM overlay was removed by washing the plate with d-PBS 100 μL/well. Monolayer cells were stained using 1 mL/well of a dye liquid (0.4% crystal violet, 1.67% ethanol in water) and then incubated for 30 min. The plate was washed gently with phosphate buffered saline (PBS) and air-dried. Positive and negative results were recorded for each well [21].

The TCID was calculated using the Reed-Muench method as follows:

1. Calculation of proportional distance (PD) between two dilutions above and below the final 50% limit:
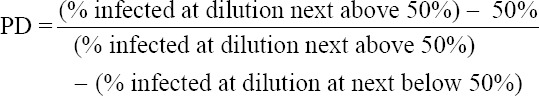
2. Calculation of the final limit of 50% (log lower dilution): the dilution close to the upper limit of 50% + PD. The reciprocal of this dilution was the virus contained in 0.1 mL of the original suspension.

### BEI preparation

BEI was acquired from BEA^®^ Sigma-Aldrich (Merck KGaA, Germany) and prepared as described by Sarachai [22]. 0.1 M BEI was prepared by dissolving 0.041 g of 2-bromo-ethylamine HBr (BEA) in 2 mL of 0.175 N NaOH. The mixture was then incubated for 1 h at 37°C. The change from BEA to BEI was followed by a decrease in pH from 12.5 to 8.5. The formation of BEI can be monitored using the pH indicator β-naphthol violet or other pH meters.

### Virus inactivation

FMDV, which had been previously cultured and whose titer was calculated using the TCID_50_ method, was diluted by adding media in MEM (−) to dilute the virus until the titer reached 10^7^/mL [15]. The virus was then inactivated with three different inactivating agents using 0.04% formaldehyde for the first treatment (T1), followed by 0.001 M BEI for the second treatment (T2), and a combination of 0.04% Formaldehyde and 0.001 M BEI for the third treatment (T3).

Absolute formaldehyde was used at concentrations of 0.04%, according to Soliman *et al*. [23]. Absol-ute formaldehyde was diluted to 1%. Inactivation was performed by adding formaldehyde to the virus suspension at a concentration of 0.04%, followed by incubation for 24 h at 37°C [13, 23, 24]. Inactivation using BEI began by adding 0.1 M BEI to the virus at a final concentration of 1% 0.001 M in a beaker, followed by incubation for 24 h at 37ºC. Inactivation using a combination of formaldehyde and BEI began by adding BEI until it reached a concentration of 0.001 M and then mixed with formaldehyde until it reached a concentration of 0.04% in a beaker. The virus was incubated for 24 h at 37°C.

Inactivation was performed for 24 h in a 5% CO_2_ incubator at 37°C [15, 23–25]. At the end of inactivation, the virus was collected and stored at −80°C after being taken as needed for further testing. BHK-21 cells were cultured on M-24 culture plates to test for viral inactivation. BHK-21 cell culture was performed with the requirement for BHK-21 cells in an M-24 culture plate to be 1.6 × 10^5^/well. After the cultured BHK-21 cells reached 80% confluence, the inactive virus was propagated into BHK-21 cells and incubated at 37°C for 1 h for virus absorption. Next, 1 mL/well of 5% MEM was added to each well. The M-24 culture plate was then incubated at 37°C in an atmosphere of 5% CO_2_. CPE was observed daily using an inverted microscope for 72 h [13, 14, 17, 23, 26].

### CPE calculation

**Figure-1:**
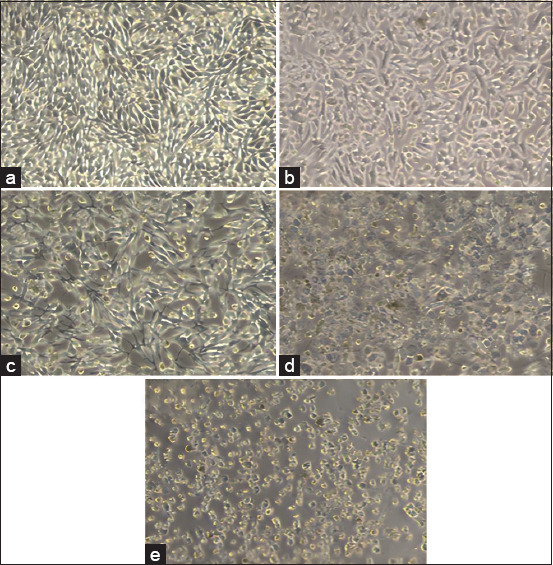
Assessment of cytopathic effects (CPE) in baby hamster kidney-21 cells after virus propagation (personal documentation using an inversion microscope at 100× magnification). (a) CPE score 0 (no CPE): Cell morphology appears normal, similar to fibroblast cells and extracellular bridges not damaged. (b) CPE score 1 (0–25% CPE): The cell shape begins to round at a mild level, and the extracellular bridges are not damaged. (c) CPE score 2 (25–50% CPE): cells round to a moderate degree, extracellular bridges begin to break down to a mild degree, and some cells fall off. (d) CPE score 3 (50–75% CPE): The majority of cells are round, extracellular bridges are damaged, and the majority of cells are loose. (e) CPE score 4 (75–100% CPE): The shapes of all cells were round, extracellular bridges were damaged, and all cells were detached from the base of the plate [17].

The infectivity of the virus was determined by observing the presence of CPE in BHK-21 cells in an M-24 culture plate that was blindly passaged thrice. If no CPE was observed from the beginning to the end of the third passage, the virus in the sample was considered non-viable and successfully inactivated [14, 27]. The percentage of CPE arising was determined using the scoring method presented in Figure-1.

### Sodium dodecyl sulfate-polyacrylamide gel electrophoresis (SDS-PAGE)

The inactivated virus was then subjected to SDS-PAGE. In the SDS-PAGE test, the gel separation concentration was 15% [28]. A total of 10 μL of sample buffer (SDS, Laemmli, β-mercaptoethanol) was mixed with 10 μL of sample or control and then heated for 5 min at 95°C to denature the protein. As a marker and control, 7 μL of the protein marker (Thermo Scientific, USA) was used [29].

Protein separation was performed via electrophoresis at 120 V and 23 mA for 2 h. The final process of serum characterization involved gel electrophoresis staining with Coomassie Brilliant Blue for 1 h, followed by a 24-hour destaining process [30, 31].

### Western blotting

The results of the SDS-PAGE test were transferred to a nitrocellulose membrane cut according to the size of the SDS-PAGE agar using the Bio-Rad Trans-Blot Turbo^®^ system (Bio-Rad Laboratories Inc., USA). (20 min, 25 V, 2.5 A).

The membrane was then blocked with blocking buffer (4% skim milk in PBS 1×) for 1.5 h at room temperature (25°C–28°C). The membrane was washed with PBS-Tween 0.05%, incubated with a 1:5 dilution of primary rabbit serum at 4°C for 24 h, and washed again with PBS-Tween 0.05%. The membrane was then incubated with HRP-labeled anti-rabbit conjugate whole immunoglobulin (Ig) G^®^ (1:10,000 dilution in PBS 1×) at room temperature (28°C–30°C) for 3 h. The membrane was then washed with diaminobenzidine (DAB) substrate^®^ (Merck, Germany) to observe the antibody and antigen binding reactions that occurred [29].

### Statistical analysis

Qualitative data from CPE in monolayer BHK-21 cells after propagation with FMDV Serotype O isolates before and after inactivation with formaldehyde, BEI, or their combination were analyzed using SPSS version 23 (IBM Corp., NY, USA). The non-parametric Kruskal–Wallis test was used to examine the relationships among all treatment groups, followed by the Mann–Whitney U test to determine the relationships among treatment groups.

The SDS-PAGE results are displayed on agar in band form. The width of the band was measured using ImageJ software (https://imagej.net/ij/) to determine the MW of the protein. After performing the measurements, the retardation factor (Rf) was obtained.

The Rf value was obtained by comparing the migration distance of the sample protein molecules with the color migration distance. Next, a standard curve was created from the standard log MW (log MW) values along the Y-axis and the standard Rf along the X-axis. The regression line equation from the curve was later used to determine the log MW value for each band. After determining the log MW value, we changed the value to anti-log to obtain each band MW value [32].

The results of Western blotting are displayed in the form of bands on a nitrocellulose membrane after reaction with the substrate to observe the binding of the specific antigen and antibody [33].

## Results

### BHK-21 cell culture

**Figure-2:**
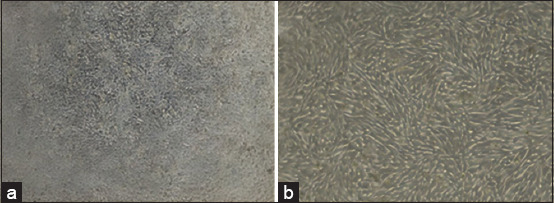
The normal appearance of baby hamster kidney-21 cells on day 3 post-inoculation was characterized by even growth, with confluency reaching 90%. The morphology of the cells appeared normal, with an elongated and tapered shape on both sides. No visible growth of bacteria or fungi was observed after viewing using an inverted microscope at (a) 40× magnification and (b) 100× magnification.

The results of this study showed that BHK-21 cells, which were inoculated into a flask using MEM with 10% FBS (MEM 10%), were successfully cultured without the visible growth of bacteria, fungi, or other toxic substances on the cells. Figure-2 shows that the cultured cells had a good morphology with an elongated and conical shape on both sides, similar to fibroblast cells, and had strong adhesion to the surface of the substrate on the flask [34].

### FMDV propagation and adaptation

**Figure-3:**
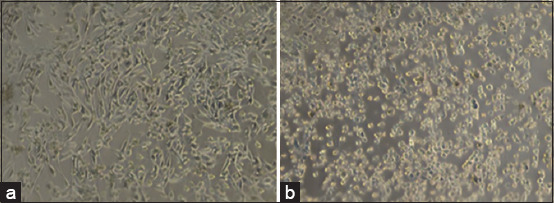
Image of baby hamster kidney (BHK)-21 cells 24 h after foot-and-mouth disease virus (FMDV) propagation (40× magnification). (a) BHK-21 cells at 24 h after FMDV propagation. (b) BHK-21 cells 72 h after FMDV propagation.

Propagation of FMDV in BHK-21 cells characterized by the appearance of CPE on the cells. The virus was then successfully adapted to BHK-21 cells by repeated passages, and the same CPE image was observed in the third passage compared with the first. All cells experienced a total CPE 72 h after FMDV propagation (Figure-3).

### TCID_50_

**Figure-4:**
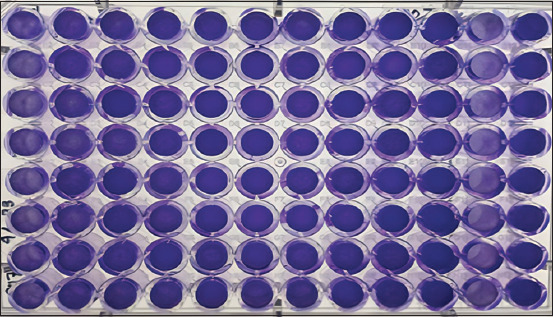
TCID_50_ assay was performed on M-96 culture plates. Dilution 10^−1^ (wells 1A-1H), dilution 10^−2^ (wells 2A-2H), dilution 10^−3^ (wells 3A-3H), dilution 10^-4^ (wells 4A-4H), dilution 10^−5^ (wells 5A-5H), 10^−6^ dilution (6A-6H well), 10^−7^ dilution (7A-7H well), 10^−8^ dilution (8A-8H well), 10^−9^ dilution (9A-9H well), dilution 10^−10^ (wells 10A-10H), positive control (wells 11A-11H), negative control (wells 12A-12H).

On the M-96 culture plate, differences were observed between BHK cells infected with FMDV and those not infected with the FMDV (Table-1). BHK-21 cells infected with FMDV experienced detachment from the bottom of the well, as shown by the bottom not being stained with crystal violet (Figure-4).

**Table-1:** TCID_50_ result analysis.

Viral dilution	Number of infected wells (+)	Number of uninfected wells (−)	Positive result ratio	% Infection (Ratio × 100%)
10^−1^	8	0	8/8	100
10^−2^	8	0	8/8	100
10^−3^	8	0	8/8	100
10^−4^	8	0	8/8	100
10^−5^	8	0	8/8	100
10^−6^	8	0	8/8	100
10^−7^	8	0	8/8	100
10^−8^	5	3	5/8	62.5
10^−9^	4	4	4/8	50
10^−10^	1	7	1/8	12.5

### Foot and mouth disease virus inactivation

The virus with a titer of 10^9^, which TCID_50_ previously tested, was diluted until it reached 10^7^ and then inactivated [15]. The data displayed for the inactivation treatment group using 0.04% formaldehyde, 0.001 M BEI, and a combination of 0.04% formaldehyde and 0.001 M BEI are shown in Table-2.

**Table-2:** CPE scores of the inactivation treatment group.

No. of passage	Day of observation	CPE score				

		Control (+ve)	Control (-ve)	T1	T2	T3
Passage-1	Day-0	0	0	0	0	0
	Day-1	1	0	0	0	0
	Day-2	1	0	0	0	0
	Day-3	2	0	0	0	0
Passage-2	Day-0	0	0	0	0	0
	Day-1	2	0	0	1	0
	Day-2	3	0	0	1	0
	Day-3	4	0	0	2	0
Passage-3	Day-0	0	0	0	0	0
	Day-1	3	0	0	2	0
	Day-2	3	0	0	3	0
	Day-3	4	0	0	4	0

T1=0.04% formaldehyde inactivation, T2=0.001 M BEI inactivation, T3=Combination of 0.04% formaldehyde and 0.001 M BEI inactivation

From the 1^st^ day (D1P1) until the 3^rd^ day of the first passage (D3P1), BHK-21 cells showed clear signs of damage similar to CPE, with the characteristic of the cell shape becoming round, followed by damage to the extracellular bridge. However, this effect disappeared on the 1^st^ day of the second passage (D1P2) and was not observed again until the 3^rd^ day (D3P3). The same type of cell damage occurred in the 0.04% formaldehyde and 0.001 M BEI groups.

In the FMDV inactivation group using BEI 0.001 M, the results from the first passage did not reveal the presence of CPE in the culture cells. However, on the 1^st^ day of the second passage (D1P2), signs of CPE began to appear, with the characteristic rounding of the shape of BHK-21 cells that had previously appeared normal. The signs of CPE also became more apparent on the 2^nd^ day (D2P2) and the second and third passage (D3P2). CPE was also observed on the 1^st^ day of the third passage (D1P3), and on the 3^rd^ day of the third passage (D3P3), the CPE score was the highest among all wells.

Statistical analysis of the CPE values in BHK-21 cells after propagation with inactive viruses revealed several similarities and differences between the control and treatment groups. A significant difference was found (p < 0.05) from D2P1 to D3P3 in the positive control group compared with the 0.04% formaldehyde group and the combination of 0.04% formaldehyde and 0.001 M BEI. These results demonstrated the success of inactivation because there was no CPE, indicating that viral infections were not observed in the treatment group. This was also confirmed by the lack of significant differences (p > 0.05) in all passages for 0.04% formaldehyde and the combination of 0.04% formaldehyde and 0.001 M BEI compared with the negative control group. In the BEI 0.001 M group, a significant difference (p < 0.05) was observed between D2P1 and D3P2 compared with the positive control group. However, in D1P3 to D3P3, there was no significant difference (p > 0.05) compared with the positive control group.

These results indicate that viral infection was not present until the 3^rd^ day of the second passage in the BEI 0.001 M group. However, virus inactivation was considered incomplete or unsuccessful on the 1^st^ day of the third passage because of the CPE effect, indicating that the virus re-infected the cultured cells. Viral infection in the 0.001 M BEI group was considered to have occurred on the 2^nd^ day of the second passage because there was a significant difference (p < 0.05) between D2P2 and D3P3 in the 0.001 M BEI group compared with the negative control group.

### SDS-PAGE

**Figure-5:**
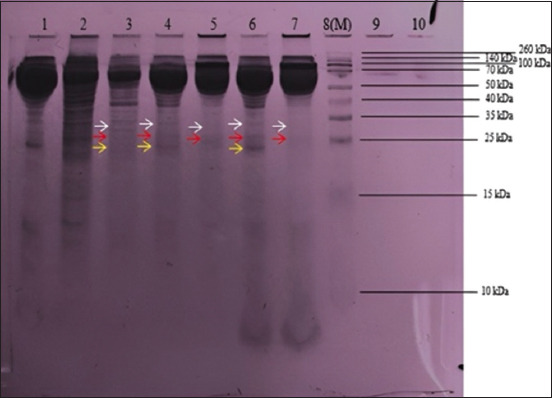
SDS-PAGE results. (1) Well 1: Minimum essential nedium (MEM, 10%). (2) Well 2: baby hamster kidney-21. (3) Well 3: pellets derived from the foot-and-mouth disease virus (FMDV). (4) Well 4: FMD virus supernatant. (5) Well 5: FMDV sample inactivated with 0.04% FA. (6) Well 6: FMDV sample inactivated with BEI 0.001 M. (7) Well 7: FMDV samples inactivated with a combination of 0.04% FA and 0.001 M BEI (8) Well 8: Marker. (9) Well 9: PBS. (10) Well 10: Empty (not filled).

Based on the assessment shown in Figure-5, bands similar to the molecular weight of FMDV (VP0 = 33 kDa, VP3 = 24 kDa, VP1 = 23 kDa) were found in the three treatment groups [35]. In the 0.04% formaldehyde treatment group, a band similar to VP0 at a molecular weight of 36 kDa and VP3 at a molecular weight of 24 kDa was observed. In the 0.001 M BEI treatment group, it can be seen that there is a band similar to VP0 at a molecular weight of 35 kDa, a band similar to VP3 at 28 kDa, and a band similar to VP1 at 23 kDa. In the 0.04% formaldehyde + 0.001 M BEI treatment group, a band similar to that of VP0 at a molecular weight of 36 kDa and similar to that of VP3 at 25 kDa was observed (Figure-5). It can be suspected that viral proteins are maintained even though inactivation is performed.

### Western blotting

**Figure-6:**
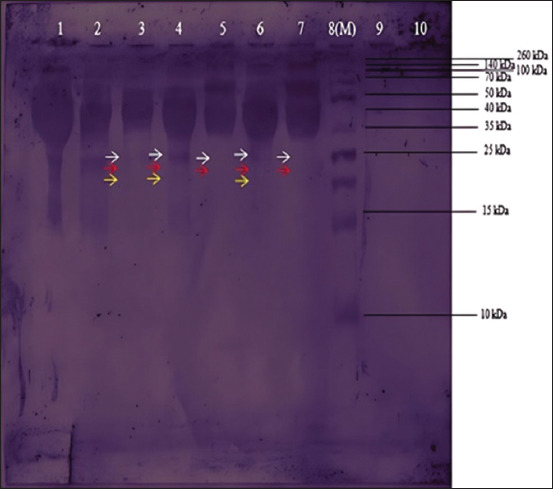
Protein transfer result in the nitrocellulose membrane. (1) Well 1: Minimum essential medium (MEM, 10%). (2) Well 2: baby hamster kidney-21. (3) Well 3: pellets derived from the foot-and-mouth disease virus (FMDV). (4) Well 4: FMD virus supernatant. (5) Well 5: FMDV sample inactivated with 0.04% FA. (6) Well 6: FMDV sample inactivated with BEI 0.001 M. (7) Well 7: FMDV samples inactivated with a combination of 0.04% FA and 0.001 M BEI (8) Well 8: marker. (9) Well 9: PBS. (10) Well 10: Empty (not filled).

The protein on the nitrocellulose membrane was FMDV because it successfully bound to antibodies from serum specific for FMDV. The presence of a band indicated the successful western blotting after the nitrocellulose membrane reacted with a DAB substrate. Figure-6 shows the bands matching the SDS-PAGE results. The expressed proteins were VP0, VP3, and VP1 [35]. VP0 is cleavaged into VP2 and VP4 during virus maturation [36].

## Discussion

Successful propagation of FMDV in BHK-21 cells is characterized by the presence of CPE. CPE appears marked by a change in the shape of normal cells to round and appear flat after 24 h of virus propagation, after which a damaged extracellular bridge is found, and eventually death and release of cells from the bottom of the flask up to 60% [17].

In the inactivation groups using 0.04% formaldehyde and a combination of 0.04% formaldehyde and BEI 0.001 M, in the first passage, there was clear cell damage similar to that of CPE with characteristic rounded cell shapes followed by extracellular bridge damage; however, in the second to the third passage, cell damage was no longer observed. The cell damage that occurred in cultured cells of both groups on the first passage was not considered CPE because these results were different from the result shown in the positive control group, which showed that the CPE caused by the virus infection remained observed from the first until the third passage, whereas, in the 0.001 M BEI group, cell damage or CPE occurred in the second to third passage.

Cell damage in the first passage of the two groups using 0.04% formaldehyde inactivation is likely to be caused by the formaldehyde substance itself. This conclusion was drawn by closely looking at first passage results for 0.04% formaldehyde and a combination of formaldehyde 0.04% and BEI 0.001 M compared with the positive control group. CPE that occurred in the D1P1 positive control group still had a low score because it took more than 24 h for the virus to cause CPE with the same characteristics of cell damage in the formaldehyde inactivation groups.

Cell damage caused by formaldehyde can be triggered by the cytotoxic effects of formaldehyde on cultured cells by inducing oxidative stress and mitochondrial dysfunction, ultimately leading to apoptosis [37]. However, the use of formaldehyde for inactivation is still permitted because of its advantages in triggering protein crosslink, as it limits the percentage of administration and removes residues after inactivation using specific methods, such as administering 2% sodium bisulfite [12, 23]. The results show that although formaldehyde caused cell damage in the first passage, no cell damage was observed in the second and third passages.

Sarkar *et al*. [13] recommended a BEI concentration of 0.001. Kim *et al*. [15] inactivated the FMD virus serotypes O (O/PAK/44/2008) and (O/IND/R2/75) at 37°C for 24 h; however, in this study, some viruses were still not inactivated. These results are consistent with a study by Sarkar *et al*. [13], which showed that the use of BEI as a single dose for virus inactivation alone has a lower inactivation speed than the combination of BEI and formaldehyde, which can inactivate the FMD virus serotype O (O/IND/R2/75) 28 times faster. The ability of formaldehyde to cross-link protein capsid stabilizes 146S virus particles, enabling BEI to more easily attack viral nucleic acids [13].

SDS-PAGE revealed that the band in the virus sample inactivated with BEI 0.001 M was the clearest. Similar results were observed in the positive control, which could be because the virus had not been optimally inactivated and possibly still replicated in cell culture. Several bands in the virus samples that were inactivated with 0.04% formaldehyde and inactivated with 0.04% formaldehyde + 0.001 M BEI appeared less precise, indicating that the protein content in the sample was not excessively high. To obtain clearer protein profiling results, concentrating the protein in the sample is needed, such as using tangential flow filtration before running the SDS-PAGE [38]. Changing the SDS-PAGE gel dye using silver nitrate (AgNO_3_) can also be performed because it is more sensitive to samples with low protein content [39].

Teng *et al*. [35] reported that the results of SDS-PAGE and western blotting identified the VP0 band at 33 kDa, VP3 at 24 kDa, and VP1 at 23 kDa. In another study by Xie *et al*. [40]. Western blotting of recombinant protein and inactivated FMDV showed bands of approximately 36 kDa corresponding to VP0 and bands of 25 kDa corresponding to VP1/VP3. Another Western blot analysis of recombinant structural proteins showed VP0 at 37 kDa, VP3 at 23 kDa, and VP1 at 23 kDa. It can be concluded that VP0 can be identified in the 33 kDa-37 kDa band and VP1/VP3 in the 23 kDa-25 kDa band, following the results of this study [41].

Apart from the FMD virus bands, several other bands were also visible. This may be because the antibodies in the serum cross-react with non-target proteins and produce false positives. Several factors, such as differences in gel-making methods, different brands of markers, and the determination of the point of the band during measurement can also cause the inaccurate location of the band in the desired area. Differences in salt content and buffer concentrations, including albumin, ovalbumin, lactate dehydrogenase, and trypsin inhibitors, can also affect protein mobility [42].

Optimizing the production of positive serum samples to avoid cross-reactions with non-target proteins can enhance the accuracy of protein determination through Western blotting. In addition, the vaccine formulation can be optimized by purification using filtration and protein chromatography methods. Using this method, impurities in the vaccine, such as host cell proteins and bovine serum albumin, can be removed so that the vaccine only contains immunogenic proteins from the virus needed to produce FMDV-specific antibodies [43].

## Conclusion

The results of this study indicate that 0.04% formaldehyde, alone or combined with 0.001 M BEI, is effective in inactivating the Serotype O FMDV while preserving its protein’s specific molecular weight. The limitation of this study was the inactivations of the virus have not yet been tested for their potency on experimental animals. Further research is warranted to investigate the inactivation kinetics of these materials, including their potency on experimental animals.

## Authors’ Contributions

FAR: Conceptualization, methodology, and research design. YK, FA, and TMT: Collected, analyzed, and interpreted data. WT, JR, YP, and KK: Contributed to the conceptualization, research design, and initial draft of the manuscript. AA, DD, FKM, HS, and SK: Contributed to the methodology, research supervision, and manuscript revisions. All authors have read, reviewed, and approved the final manuscript.
